# Effect of Weave and Weft Type on Mechanical and Comfort Properties of Hemp–Linen Fabrics

**DOI:** 10.3390/ma17071650

**Published:** 2024-04-03

**Authors:** Simona Vasile, Sofie Vermeire, Katrien Vandepitte, Veronique Troch, Alexandra De Raeve

**Affiliations:** 1 Fashion and Textiles Innovation Lab (FTILab+), HOGENT University of Applied Sciences and Arts, 9051 Ghent, Belgium; sofie.vermeire@hogent.be (S.V.); alexandra.deraeve@hogent.be (A.D.R.); 2 AgroFoodNature, HOGENT University of Applied Sciences and Arts, 9051 Ghent, Belgium

**Keywords:** hemp yarns, flax yarns, fabric weave, mechanical properties, comfort properties

## Abstract

In this study the influence of fabric weave (plain, twill, and panama) and weft type (flax and hemp yarns) on selected mechanical and comfort properties of six fabrics was analyzed. The results showed that tear and abrasion properties were most affected by the weave. The tensile properties of the linen fabrics were not significantly different when weft hemp yarns were used instead of flax. Fabrics with the same weave seemed to be equally resilient to abrasion regardless of the type of weft. By contrast, the hemp weft yarns favorized the physical and comfort properties of the investigated fabrics. For the same weave, the hemp–linen fabrics were slightly lighter and exhibited lower bulk density, significantly larger air permeability, and improved moisture management properties. Although the results of maximum thermal flux (Qmax) suggested a cooler sensation of the linen fabrics with panama and twill, the hemp–linen fabric with a plain weave seemed to be the optimal choice when a cool sensation was desired. Higher thermal conductivity values also suggested slightly better heat transfer properties of the hemp–linen fabrics, and these were significantly influenced by the weave type. The results clearly indicated the advantages of using hemp for improving physical and specific comfort properties of linen fabrics.

## 1. Introduction

Hemp fiber has served humanity for thousands of years to create textile fabrics, ropes, yarns, rugs, and canvases. The 1990s marked the renewal of hemp cultivation, about 60 years after its prohibition. In most European countries, the current upper legal limit for cultivation of industrial hemp (*Cannabis sativa* L.) for fiber and seeds production is 0.3% THC on dry basis, which led to a restriction of a number of varieties available for cultivation [1].

Recent knowledge and developments in hemp varieties, cultivation [2], and processing are expected to boost the employment of hemp in the textile sector, as a sustainable fiber complementary to flax, possible to process on traditional flax technology [3]. As a natural and environmentally friendly crop, hemp fiber is an important raw material for the textile sector due to its excellent moisture absorption and release properties, air permeability, warmth retention, cold and warm sense, and high temperature resistance, among others [4].

Hemp and flax belong to the bast cellulosic fibers, along with kenaf, ramie, jute, and nettles [5], and their differentiation is often controversial as they are very similar fibers in many aspects [6]. In general, hemp fibers are coarser [7] and have a less supple handle than flax, but they exhibit similar high tensile properties [5], low elongation, and high moisture absorption [5,6].

Although the cultivation of flax and hemp and the use of their fibers have already been described in ancient times, their importance has declined during the past few centuries as cotton has been favored. Among natural fibers, such as cotton, silk, wool, flax, hemp, etc., cotton is the one that takes up the highest percentage in the textile market [8]. Hemp is more durable than cotton and requires half the amount of water and land that it takes to grow cotton, whereas the crop can be harvested in 90 days. It is also able to be grown without the use of any herbicides and pesticides on smaller areas of land and can be used to create durable, sustainable, renewable, and flame-retardant fabrics [8].

Several studies have shown the environmental advantages of bast fibers such as hemp and flax compared with cotton. For instance, the study of Turunen from 2007 [9] compared the environmental impacts of three hemp yarn production scenarios and a flax yarn scenario and showed that the scenarios were very similar except for pesticide use (higher for flax) and direct water use (higher for hemp). For the crop production stage, hemp performed clearly better than cotton with respect to pesticide use and water use, but during fiber processing and yarn production stages, hemp required more energy. A more recent study [8] investigated the group of bast fibers (including jute, flax, hemp, and stinging nettles), as a sustainable and economic alternative to cotton and organic cotton. Hemp has proven promising; however, legal and regulatory issues, together with the lack of supply due to its infancy in the product market, block its further exploitation in the market of fibers [8]. Similarly, Schumacher et al., 2020 [10] looked at the yield and costs of agricultural activities and provided evidence that hemp fiber is a valuable sustainable alternative to cotton.

Hemp fibers’ numerous applications in textiles include cushion covers, blankets, shoes, socks, ropes, yarns, rugs, home textiles, etc. [1]. Hemp textiles are durable, breathable, versatile, and biodegradable, having strong thermal qualities, meaning they keep the wearer cool in the summer and warm in the winter. Traditional hemp textiles were too coarse for apparel, but improved techniques allow the creation of lighter and softer textures to be used for clothing such as jeans and sportswear, either in the form of 100% hemp fabric or blended with cotton, wool, flax, or synthetics [11]. Currently, clothing is a high-profile market for hemp. Preference for hemp textiles in summer is often associated with their excellent hygienic properties. Hemp fibers are also more resistant to weather and ultraviolet rays than cotton and silk [1], and nowadays, hemp can be used to make a variety of similar but more durable fabrics than cotton [1].

Linen fabrics are valued for their smoothness, silky gloss, durability, comfort, and easily recognizable, specific appearance that denotes their natural origins [11]. Linen also has a wide variety of applications in interiors, from upholstery fabrics to curtains, and shorter flax fibers produce heavier yarns, which are suitable for kitchen towels, sails, tents, and canvas. Because linen fabric is soft, does not lint, can be washed at high temperatures, and has a great capacity for absorbing moisture, it is a suitable fabric for bathroom and table linen [11]. Linen fabrics makes good bedding fabrics [12], and garments made of linen are valued for their exceptional coolness and freshness in hot weather. As compared with cotton, linen is stronger and is a better heat conductor [13]. Nevertheless, linen is less elastic than cotton and creases easily. The main problems of linen textiles are their roughness, stiffness, and wrinkling tendency, and different finishing processes are necessary [13] to improve these drawbacks.

Linen and hemp are blended with other compatible natural and manmade fibers to achieve various structural and functional properties and also to reduce costs. Linen fabrics were found to be tougher than cotton but viscose, and cotton improves their handle (fabric touch) [14]. Hemp and flax blended denim fabrics had better moisture comfort properties and thermal resistance than the cotton reference denim fabrics, and their tactile comfort properties were within acceptable limits [15]. Cotton enhanced the air permeability, water absorption, and drying rate of the flax and denim fabrics [16].

Constructional parameters greatly influence various fabric properties. The impact of fabric weave on various physical, mechanical, thermo-physiological, and comfort properties was summarized in a recent review paper [17]. Other studies highlighted the effect of weave on the thermo-physiological properties of cotton fabrics [18], bending rigidity [19], and tensile, bursting, and impact strengths of cellular woven fabrics [20], as well as on the tear strength of woven fabrics [21]. Weave and weft yarn type affect dimensional stability, air permeability, water absorption, abrasion, and bending rigidity of cotton–bamboo woven fabric [22], whereas weave and yarn density seem to impact less than yarn type, the force, and elongation at break [23].

Concluding the above studies, there is a clear need to complement the conventional fibers with more sustainable, high-quality fibers like hemp in mass consumption clothing products. With the resurgence of hemp in the clothing and textile industry, there is a need to complement the existing studies [16,17,24,25,26] dealing with hemp fabrics and the effect of constructional parameters on various fabric properties. Therefore, this study investigated the effects of fabric weave and weft type (hemp versus flax) on selected mechanical and comfort properties of hemp–linen fabrics. The study hypothesized that fabric weave would significantly affect both mechanical and comfort properties, whereas the hemp weft yarn would potentially improve the comfort properties of the linen fabrics.

## 2. Materials and Methods

### 2.1. Fabric Production

Six fabrics were prepared using a Dornier industrial weaving loom (DORNIER GmbH, Lindau, Germany) of 300 cm width equipped with 6 shafts and two rapiers and accommodated with a warp beam consisting of bleached bio-flax Nm 26/1 with 24 ends/cm. The six fabrics were differentiated by the type of weft (hemp or flax yarn) and fabric weave (plain, panama, and twill), as shown in Table 1. Ecru bio-flax yarns Nm 24/1 were used in the weft direction (FLAX PLAIN, FLAX PAN, and FLAX TWILL) or bleached hemp yarns Nm 26/1 (HEMP PLAIN, HEMP PAN, and HEMP TWILL).

### 2.2. Characterization of the Fabrics

#### 2.2.1. Fabric Physical Properties

The fabric mass per unit area (g·m^−2^) was assessed according to ISO 3801-1977 [27]. The mean value of ten square specimens of 100 cm^2^ weighted with an accuracy of 0.001 g was calculated and expressed to the nearest gram.

The thickness of the fabrics (mm) was assessed according to ISO 5084-1996 [28] under a pressure of 1 kPa using a Hess thickness gauge model HDM-3 (Richard Hess MBV GmbH, Sonsbeck, Germany) with an accuracy of 0.01 mm. The mean value of ten measurements was expressed to the nearest 0.1 mm.

The bulk density of the fabrics (kg·m^−3^) was calculated as ratio of fabric mass per unit area (g·m^−2^) and thickness (mm).

The relative porosity *P* (%) was calculated using the equation below:

(1) $$P=\left(1-\frac{m}{\rho \times {d\cdot 10}^{3}}\right)\times 100$$

where *m* is the fabric mass per unit area (g·m^−2^), *ρ* is the fiber density (g·m^−3^), and *d* is the fabric thickness (mm). The fiber density *ρ* of the three hybrid flax–hemp fabrics was calculated using the next equation:

*ρ* = *a*_1_ × *ρ*_1_ + *a*_2_ × *ρ*_2_ (2)

where *a*_1,2_ and *ρ*_1,2_ represent the percentage hemp–flax and hemp (1.47 g·cm^−3^) and flax (1.54 g·cm^−3^) fiber density, respectively [29]. The hemp and flax percentages were approximated as being equal (0.5), based on the comparable count of the yarns and yarn density in weft and warp direction.

#### 2.2.2. Fabric Mechanical Properties

Tensile, bending, tear, and abrasion properties of the six fabrics were determined as described below.

The tensile properties were measured in dry state following the ISO 13934-1:2013 [30] strip method. A tensile tester was used with a gauge of 200 mm and a constant rate of extension (CRE) of 100 mm/min. Five fabric strips (50 × 300 mm) were tested in both fabric directions (weft and warp). Mean values and standard deviations (STDEVs) of the maximum load and elongation at break were calculated.

The bending properties were determined according to DIN 53864 [31] (the Schlenker method). Five samples of 2 × 5 cm were tested in both fabric directions, and the mean values and STDEVs were calculated for the fabric bending strength.

The fabric tear properties were initially assessed according to ISO 13937-1:2000 [32] (the ballistic pendulum Elmendorf). This attempt failed as the force needed to tear the specimen exceeded the maximum force exerted by the instrument. The tear properties were eventually assessed by the single tear method ISO 13937-2:2000 [33], using a tensile tester and a trouser-shaped legs specimen. For each fabric direction, five rectangular specimens were prepared of 200 × 50 mm, including a slit of 100 mm length in the shorter edge. The sample was torn with a rate of extension of 100 mm/min on a total tear length of 75 mm, and the arithmetic mean for all peaks recorded over the calculation area was calculated. A peak suitable for calculation was characterized by a 10% rising/falling of force. The calculation area was considered from 10 mm after the start of the test to 10 mm before the test’s end.

The abrasion properties were determined according to ISO 12947-2:2016 [34], using a Martindale apparatus under a load of 12 kPa until 6000 cycles, with inspection intervals of 1000 cycles. The number of abrasion cycles that caused the complete breakdown of the first two yarns was registered.

#### 2.2.3. Fabric Comfort Properties

The air permeability was determined as the velocity (mm/s) of an air flow passing perpendicularly through a test specimen under specified conditions (ISO 9237-1995 [35]). Air permeability was measured at a pressure drop of 100 N on a fabric test surface of 20 cm^2^ using an air permeability apparatus (EMI Development). The mean value and STDEV of five specimens were calculated for each fabric.

The moisture management properties of the six fabrics were assessed by a moisture management tester (MMT) according to AATCC 195-2011. The MMT measured several indices, such as the wetting time (WT), maximum wetted radius (MWR), spreading speed (SS), and absorption rate (AR) on the top and bottom surfaces of the fabrics and calculated the accumulative one-way transport capability (R) and overall moisture management capacity (OMMC). A grading from 1 to 5 (low to high) was applied to all indices. The mean value and STDEV were calculated for five specimens of 8 cm × 8 cm.

The thermal properties were determined using the thermal module of the fabric touch tester (FTT) apparatus. The specimen was placed between two plates, and when a 10 °C temperature difference was achieved between the plates, the upper plate moved downward to compress the specimen and then returned upward (recovery). The maximum thermal flux Qmax (W/m^2^) measured during the compression phase (about half of the total 120 s testing period) and the calculated thermal conductivity TCC (W/m°K) were reported, as the mean value of five square specimens of 11 cm × 11 cm.

All specimens were conditioned prior to testing for a period of 24 h, at 20 ± 2 °C and 65 ± 4% relative humidity (ISO 139:2005 [36]) in a conditioning chamber. A paired *t*-test (two samples with unequal variances) was used to identify statistically significant differences (*p* < 0.05) between the fabric properties depending on the test direction (weft–warp) or type of weft yarn (hemp–flax). The influence of the three weaves on the mechanical and comfort properties was statistically evaluated using one-way variance analyses (ANOVA).

## 3. Results and Discussions

### 3.1. Fabric Physical Properties

**Figure 1:**
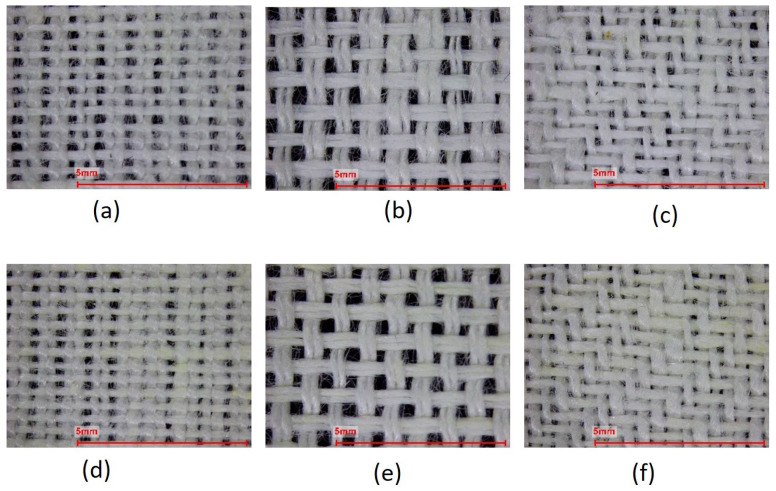
Digital images (magnification ×15) of fabric HEMP PLAIN (**a**), HEMP PAN (**b**), HEMP TWILL (**c**), FLAX PLAIN (**d**), FLAX PAN (**e**), and FLAX TWILL (**f**).

The structural and physical properties are shown in Table 2, and Figure 1 displays the microscopic images of the six fabrics. The fabric mass varied between approximately 181 g·m^−2^ and 199 g·m^−2^. For the same weave, the linen fabrics were slightly heavier than the hemp–linen fabrics, and given the similar fabric construction, this difference could be attributed to the different density of flax (1.54 g·cm^−3^) and hemp fibers (1.47 g·cm^−3^) [29]. Among the considered weaves and for the same weave type, the plain fabrics (HEMP PLAIN and FLAX PLAIN) were slightly heavier. The fabrics had comparable thicknesses that varied between 0.51 mm and 0.55 mm. The fabric bulk densities varied between 333 kg·m^−3^ and 390 kg·m^−3^, with the linen fabrics having the highest value among the fabrics with same weave and weft type. The plain fabrics (HEMP PLAIN and FLAX PLAIN) were more compact and exhibited the highest bulk density and the lowest porosity, when compared with the panama (HEMP PAN and FLAX PAN) and twill (HEMP TWILL and FLAX TWILL) fabrics. This can also be clearly deducted when comparing Figure 1a,b versus Figure 1c–f (all magnifications ×15).

### 3.2. Influence of Weave and Weft Type on Fabric Mechanical Properties

#### 3.2.1. Tensile Properties

The mechanical properties of a woven fabric are influenced by various factors such as the strength of the yarn, the setting of the fabric, and the function of the coefficient of strength of the yarn. It has been proven that an increase in the yarn strength leads to fabrics with elevated strength [17].

**Figure 2:**
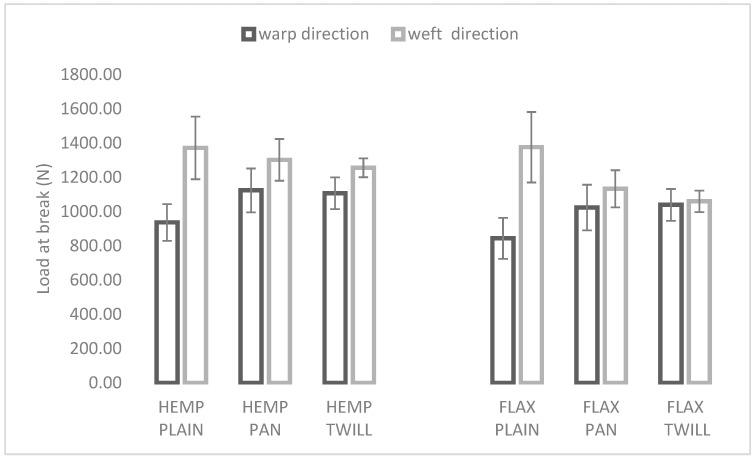
Influence of weave and weft type on load at break of the hemp blended (**left**) and flax (**right**) fabrics.

In Figure 2 the load at break of the hemp–linen (left) and linen (right) fabrics is shown in both the weft and warp directions.

The graph illustrates a similar effect of weave on both groups of linen (right) and hemp–linen fabrics (left). In the cases of both the linen fabrics and hemp–linen fabrics, comparable results were obtained for the load at break in the cases of the twill and panama fabric.

All six fabrics had higher values of load at break in the weft direction, but these direction-based differences were statistically significant only in the case of the plain weaves (HEMP PLAIN and FLAX PLAIN) as indicated in Table 3. A clear influence of the weft type on the load at break can be seen in Figure 2. For the same weave and direction, the absolute values were higher in the case of the hemp–linen fabrics. These larger values could be related to the strength of the hemp yarns used in the weft direction (4249.2 ± 316.7 cN), as compared with the flax weft (3998.2 ± 519 cN) and warp (3881.9 ± 213.1 cN) yarns. In the warp direction, the differences between the load at break of linen and hemp–linen fabrics with different weaves were not statistically significant. However, in the weft direction, the hemp–linen fabrics with twill and panama weaves were significantly stronger than the linen fabrics, as can be seen in Table 3.

**Figure 3:**
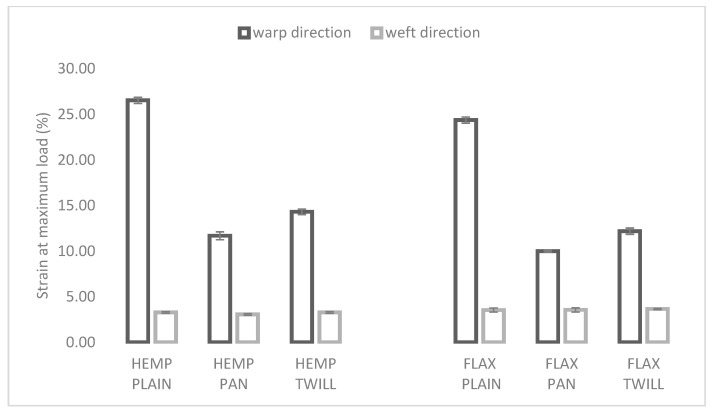
Influence of weave and weft type on elongation at break of the hemp-blended (**left**) and flax (**right**) fabrics.

In Figure 3 the elongation at maximum load is shown for the hemp–linen (left) and linen (right) fabrics with different weaves. The influence of the weave can be clearly seen in the warp direction, where the plain weave was significantly more elastic than the twill and panama weaves, in both the cases of linen and hemp-blended fabrics. As shown in Table 3, all six fabrics had significantly lower elongation in the weft direction, regardless of the weave and weft type. The weft type significantly influenced fabric elongation in both directions. For the same weave, hemp–linen fabrics were more elastic in the warp direction and the linen fabrics in weft direction, as can be seen in Figure 3. The hemp fibers were less elastic (1.6%) at break than the flax fibers (1–4%) [29]. The flax weft (2.97 ± 0.53%) and warp (3.65 ± 0.84%) yarns used in this study were slightly more elastic than the hemp weft yarns (2.74 ± 0.28%).

The results of our study are aligned with a previous study [24] that showed lower breaking strength and higher elongation of hemp plain fabrics for upholstery than the twill and modified twill fabrics, in both fabric directions. Similarly, a lower tensile strength was reported for plain than for twill and satin weaves, which was explained by the greater effect of contact friction, crimp, and binding on plain weave [23]. The same study also reported a higher elongation in plain weave, due to the higher interlacement and the crimps present in a plain weave structure.

#### 3.2.2. Bending Properties

**Figure 4:**
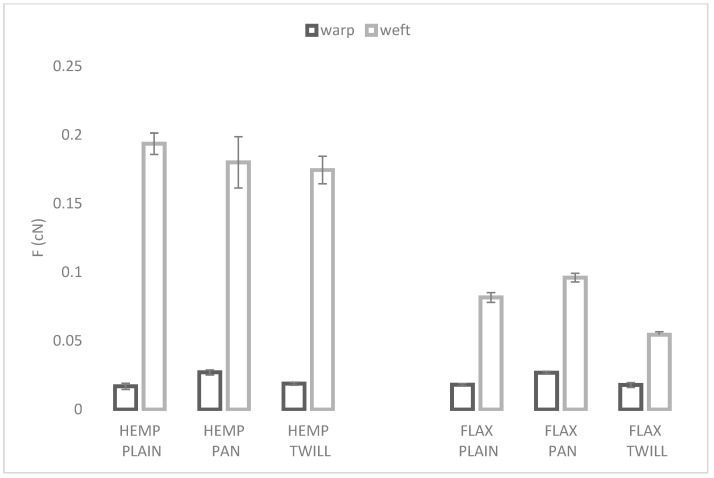
Bending strength of the fabrics in the warp and weft directions.

The bending strengths of the six fabrics are displayed in Figure 4. The results clearly show that the bending properties were direction-dependent, and the forces needed to bend any of the fabrics in the weft direction were significantly higher than in the warp direction, as listed in Table 3. The influence of the weave on the bending properties of linen and hemp–linen fabrics was consistent only in the warp direction. For the linen fabrics, panama weave seemed to require the highest load to bend. Hemp-blended fabrics exhibited the same trend only in the warp direction, while plain fabrics seemed more resilient to bending in the weft direction. Nevertheless, the large STDEV for the hemp-blended fabrics with panama weave could explain this inconsistency. Our results were partially in line with a study [19] that investigated the effects of various weaves on the bending properties of five woven fabrics and attributed a decrease in bending rigidity to an increase in yarn floats [19]. For the same weft type, another study [22] showed that the bending rigidity was highest for plain weave, followed by panama and twill. In our study, we noticed this trend only in the weft direction for the hemp–linen fabrics.

The influence of the weft type is clearly visible in Figure 4. For the same weave, no significant different load was required to bend the plain (*p* = 0.33), the panama (*p* = 0.84), or the twill (*p* = 0.32) linen–hemp or linen fabrics in the warp direction, as is shown in Table 3. However, a significantly higher load was necessary to bend the hemp–linen fabrics in the weft direction regardless of their weave, and this could be attributed to the less elastic and supple hemp yarns. A recent study [22] compared fabrics with four weaves and various bamboo–cotton weft yarns and concluded that weave type accounted for about 80% of the bending rigidity and the weft type accounted for around 16% [22]. In our study, a significant influence of the weave on the bending of linen fabrics was found and of hemp–linen fabrics in the warp direction.

#### 3.2.3. Tear Properties

**Figure 5:**
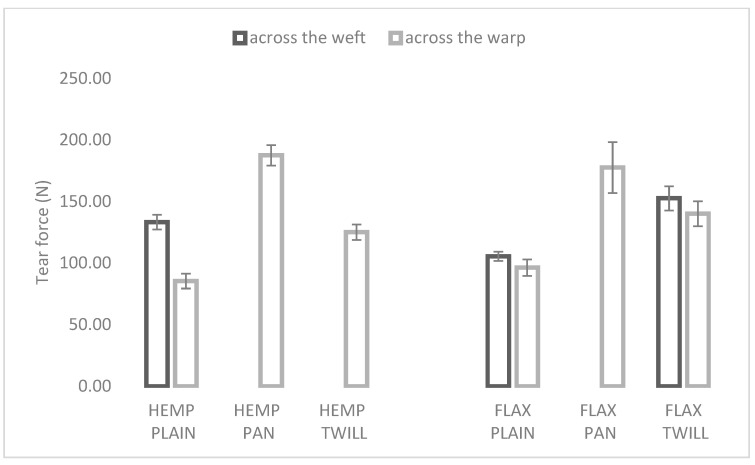
Tear force of the hemp-blended (**left**) and flax (**right**) fabrics with various weaves.

Tearing can be described as the sequential breakage of yarns or groups of yarns along a line through a fabric. It is one of the most common types of failure in textile materials and, in many cases, serves to terminate their useful life. The tearing strength is often used to give a reasonably direct assessment of serviceability, more than the tensile strength, and a fabric with low tearing strength is generally an inferior product. Unlike in the case of tensile loading where all the yarns in the direction of loading share the load, in tear loading only one, two, or at most a few yarns share the load [37]. Figure 5 shows the tear force of the analyzed fabrics. Within the test period, all five specimens of each fabric tore in the warp direction. By contrast, in the weft direction, five specimens of FLAX PLAIN, three specimens of FLAX TWILL and HEMP PLAIN, and no specimen of HEMP PAN, HEMP TWILL, and FLAX PAN tore across this direction.

The weave type had a significant effect on the tear force. The lowest tear force was registered for plain weave in both the cases of hemp–linen and linen fabrics. This was in agreement with other research [37], which found the lowest tearing strength for the plain fabrics among several investigated weaves (warp rib, weft rib, ripstop, and plain weave) and explained that by the loss of mobility and tight construction of the plain weave, which restricted the movement of the yarns. By contrast, loose and open constructions such as rib and basket weaves allow yarns to move and group together, and therefore, their tear strength is high [37]. Similar behavior was noticed in this study. Furthermore, another study [38] stated that the greater the number of yarn intersections in the direction of tearing, the more the tightness of the structure, the less freedom of yarn to slide, and the less yarn sharing to resist the tearing. Hemp twill and modified twill fabrics for furnishing applications also exhibited higher tearing strength than plain fabrics in both directions [24]. The fabric firmness, density, and friction of the yarns affected the tear resistance of woven shirting fabrics with different yarns reversely [39]. Fabrics with longer floats had higher tear strength in comparison with the fabrics having no floats like plain weave [21], which was also observed in our study.

#### 3.2.4. Abrasion Properties

The surface of the six fabrics was subjected to abrasion, under a pressure of 12 kPa as required for fabrics intended for workwear, upholstery, bed linen, and fabrics for technical use. For each fabric, four specimens were tested until 6000 cycles, with evaluation intervals of 1000 cycles. The test was stopped when two threads completely broke, and the results are shown in Table 4. It was observed that fabrics with the same weave seemed to be equally resilient to abrasion, regardless of the type of weft. This was expected because the abrasion applied was not direction-dependent.

Nevertheless, a clear influence of the weave could be noticed for any of the linen nor hemp-blended fabrics. Among the six fabrics analyzed, the panama weave was clearly the most sensitive to abrasion when compared with the plain and especially the twill weaves. This is partially in line with previous research [22], which observed that the effect of the weave type on abrasion resistance was stronger than the fiber type and reported more abrasion in the twill and satin weaves than in plain and panama, attributed to the longer floating yarns of the former two weaves. Among the three types of weaves investigated (i.e., plain, twill, and modified twill), the plain hemp fabrics for furniture had the best abrasion resistance, suggesting that the higher number of interfacings and absence of the floating yarns resulted in better resistance to abrasion [24].

### 3.3. Influence of Weave and Weft Type on Fabric Comfort Properties

Comfort is one the most important aspects for clothing and depends on the fabric transmission properties. Some comfort-related transmission properties of fabrics include air permeability, moisture management properties, thermal conductivity, water vapor permeability, and wicking properties [40].

#### 3.3.1. Air Permeability

The air permeability is a measure of how well a fabric allows air to pass through. Air permeability plays a significant role in textiles for clothing due to its influence on physiological comfort but is also very important in technical textiles, especially for filtration, automotive airbags, parachutes, etc. [41].

**Figure 6:**
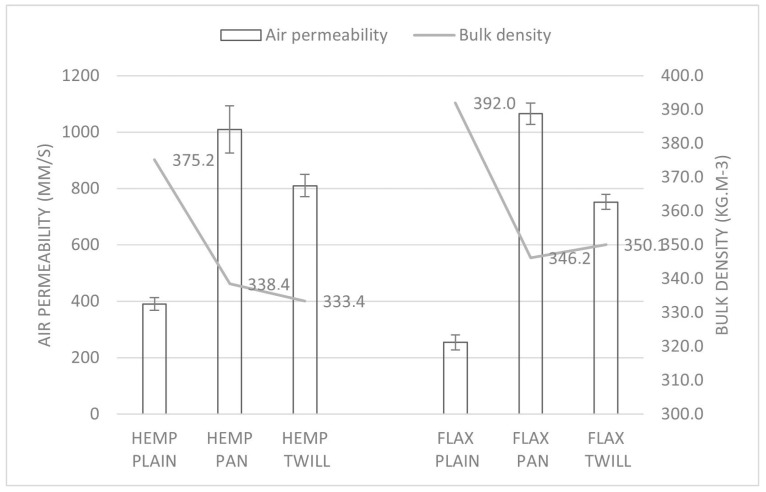
Air permeability and bulk density of the six fabrics.

**Figure 7:**
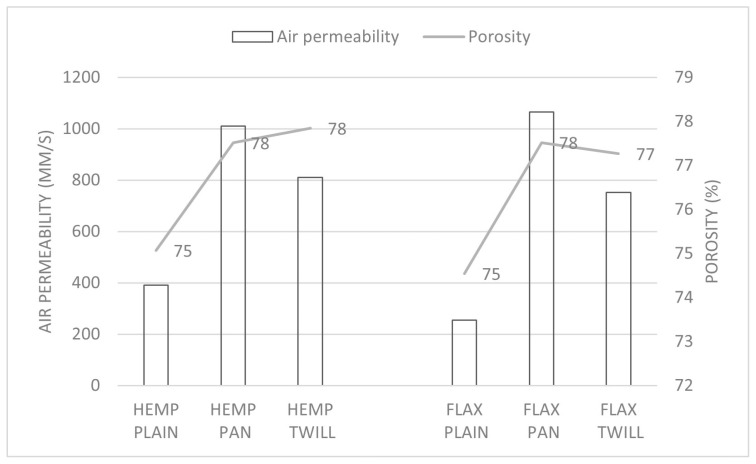
Air permeability and porosity of the six fabrics.

The values registered for the air permeability are shown in Figure 6 and Figure 7 as a function of bulk density and porosity, respectively. The lowest air permeability was observed for the hemp–linen and linen fabrics with plain weave (HEMP PLAIN and FLAX PLAIN), which were the least porous (both 75%). On the other hand, the fabrics HEMP PAN and FLAX PAN with the highest porosity (both 78%) exhibited the highest air permeability (Figure 7).

The influence of the weave on the air permeability is also clear from Figure 6 and Figure 7, which display significant different air permeabilities depending on the weave type in both groups of fabrics. For the same weave, the air permeability of the plain and twill fabrics significantly increased (as shown in Table 5) after replacing the weft flax yarn by a hemp yarn of comparable linear density.

A high positive correlation was found between the air permeability and porosity (Pearson coefficient 0.92) and a negative correlation with the bulk density (Pearson coefficient −0.9) and weight (Pearson coefficient −0.8) of the fabrics. Despite a comparable porosity, the hemp–linen fabrics were lighter and had a lower bulk density, which could be attributed to the lower density of the hemp fibers [29] and also to the slightly finer weft hemp yarn (Nm 26), compared with the flax weft yarn (Nm 24). A recent comprehensive literature review [42] also stated that air permeability decreases with increased fabric density and weight, depending on the fabric materials, blend ratio, and fabric structure. Our findings were in agreement with another study [43], which found the plain weaves less permeable than the twill and satin weaves. The air permeability of cotton–bamboo fabrics in satin weave (with the low number of intersections between weft and warp yarns) was the highest, as compared with the plain weave, with the highest number of intersections [22]. Moreover, the air permeability decreased as the weight increased [22], which was also observed in our study. The fabric weave and picking sequence had a significant effect on the fabric air permeability. Better air permeability was registered for 3/1 twill fabrics with simultaneous three pick insertion as compared with plain fabrics and those with double or single picking sequences [44]. Fabric air permeability largely depends on the weight of the fabric, the thickness and porosity [40], the structural properties, and the interactions thereof [41]. A recent review [17] stated that the fabric density, warp and weft densities, and weave are the key structural characteristics that affect fabric air permeability.

#### 3.3.2. Moisture Management Properties

The moisture management properties of the six fabrics are summarized in Table 6. During testing, distinctive behavior was noticed for specimens within the same fabric type, which was reflected by the high variability (STDEV) of some indices, for hemp–linen fabrics in particular.

In general, for the same weave, hemp–linen fabrics exhibited higher values than the linen fabrics for the top/bottom absorption rates, maximum wetted radius, and spreading speed. The absorption rate reflects the ability of the fabric top and bottom surfaces to absorb moisture. The maximum wetted radius and spreading speed reflect the moisture spreading ability and the spreading speed to reach the maximum wetted radius, respectively. The spreading speed is an indication of a fabric’s ability to dry quickly. These results suggest better management properties of the hemp–linen fabrics.

The accumulative one-way transport capacity (R) is the difference in the cumulative moisture content between the top and bottom surfaces of the fabric and is a direct indication of the fabric’s ability to allow liquid that wets the inner surface to move toward the outer surface. The R-index is particularly linked to the comfort as any trapped moisture in the inner surface over a long time period results in a great physiological discomfort to the wearer. For the same weave, despite the large variations within most of the fabrics, lower R-index values were noticed for the hemp–linen fabrics.

The overall moisture management capability (OMMC) reflects the overall capability of a fabric to transport liquid moisture and is calculated by combining three measured attributes of performance: the liquid moisture bottom absorption rate, the accumulative one-way liquid transport capability (R), and the bottom moisture spreading speed. The OMMC indices of the six fabrics were comparable, with values between 0.5 and 0.7. Two hemp–linen fabrics (HEMP PLAIN and HEMP TWILL) were classified as moisture management fabrics, while HEMP PAN, FLAX PLAIN, FLAX TWILL, and FLAX PAN fabrics were classified as water penetration fabrics. Except for FLAX PLAIN, these fabrics had a more open structure and exhibited the highest porosity, which could explain the quick passing of water droplets from the top to the bottom side of the fabric. A previous study [44] linked OMMC with the weave float and found that the moisture management capability of twill fabrics 3/1 was greater than that of plain fabrics due to their better wicking property. In our study a similar trend was observed, suggesting better moisture management properties of the fabrics with twill and panama weaves, regardless of the weft type. Another study [44] also revealed that the fabric weave and picking sequence had a significant effect on the fabric wetting time, water spreading speed, and also its air permeability. A recent comprehensive literature review [42] highlighted that absorbency was significantly correlated with the OMMC and therefore recommended the first as a good proxy for OMMC, which required more sophisticated measurements. OMMC correlated weakly with the fabric thickness [42].

#### 3.3.3. Thermal Properties

**Figure 8:**
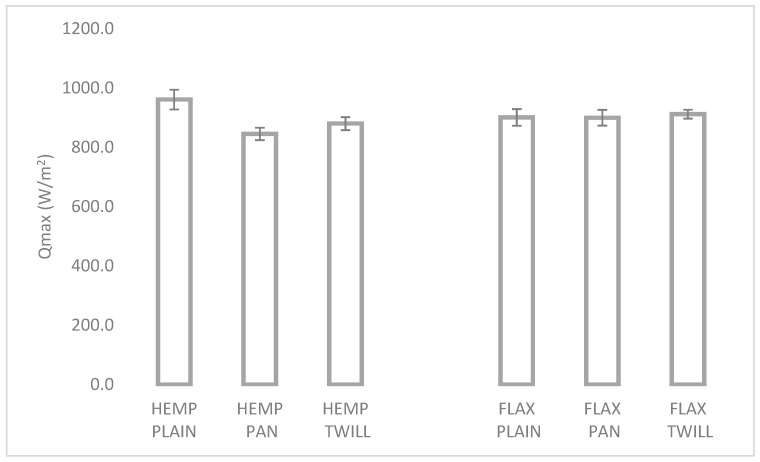
Maximum thermal flux Qmax of the six fabrics.

The Qmax index indicates the coldness and warmth feeling that affects the sensation of coldness or warmth of skin touching fabric. It is determined by the heat loss from the skin to the fabric [45]. A higher Qmax value means a cooler initial feeling when touching the fabric. Figure 8 shows the values of the maximum thermal flux Qmax of the linen (right) and hemp–linen (left) fabrics measured by the fabric touch tester (FTT) and shows values between 845 W/m^2^ (HEMP PAN) and 961 W/m^2^ (HEMP PLAIN).

The absolute values measured in this study were aligned with the study [45] that reported Qmax values (measured by KES-F7 device) of 0.093 and 0.103 W/cm^2^, for cotton knitted fabrics with lower bulk densities than in this study. The type of weave led to significant different Qmax values in the case of hemp–linen fabrics, while these were comparable (around 900 W/m^2^) among the three linen fabrics. The linen fabrics with panama and twill weave exhibited significantly higher Qmax (as shown in Table 5), which suggested that they would feel cooler at the first touch than the hemp–linen fabrics. Nevertheless, the HEMP PLAIN fabric had a significantly higher Qmax than FLAX PLAIN and therefore seemed the optimal choice among the six fabrics investigated, when a cool sensation was desired.

**Figure 9:**
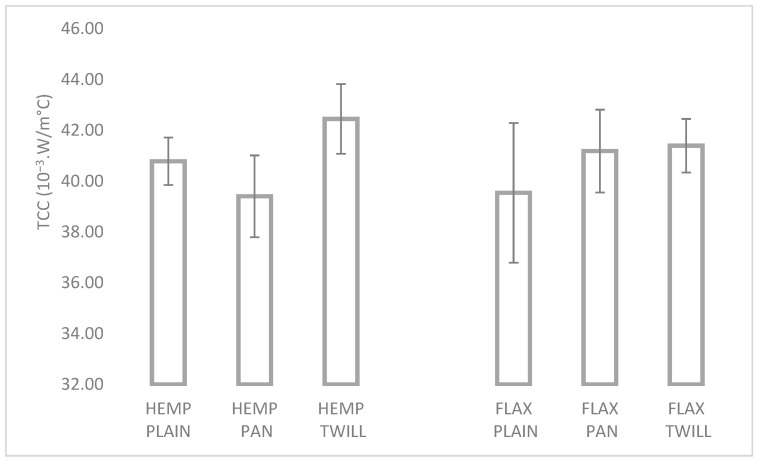
Thermal conductivity of the six fabrics.

Thermal conductivity refers to the ability to transfer heat. A fabric with a high thermal conductivity permits the transfer of heat from a hot side (i.e., in contact with the human body) to a cooler side, such as the air on the other side of clothing. The thermal conductivities of textile structures generally level from 0.033 to 0.10 W/m·K [46]. The same study investigated woven wool and wool–polyester fabrics and reported thermal conductivities in the range of 0.043–0.049 W/m·K, while values of 0.022 W/m·K, 0.026 W/m·K, and 0.034 W/m·K were reported for knitted fabrics of hemp, cotton, and hemp–cotton [47]. The values of thermal conductivities measured during the compression period (TCC) are shown in Figure 9 and varied in the range 0.039 W/m°C (HEMP PAN) to 0.042 W/m°C (HEMP TWILL); the higher the TCC value, the better the thermal conductivity of the fabric.

The influence of the weft type is clearly visible in Figure 9. Although higher values of the TCC can be observed for the hemp–linen fabrics with plain and twill weave, the differences were not significant for any of the weaves, as shown in Table 5. Variations of the TCC due to the weave can be noticed in both group of fabrics, but they were significant only in the case of the hemp–linen fabrics.

## 4. Conclusions

This study investigated the effects of weave and weft type on several mechanical and comfort properties of six fabrics and confirmed the research hypotheses. The use of hemp warp yarns had clear advantages and seemed to favor mainly the physical and the comfort properties of the blended fabrics. For the same weave, the hemp–linen fabrics had slightly lower mass per unit area and lower bulk density than the linen fabrics. Furthermore, their air permeability significantly increased, and their overall moisture management properties improved. Hemp–linen fabrics exhibited higher values of moisture absorption rates, maximum wetted radius, and spreading speed, which suggested their potential to dry faster. Although the results of Qmax suggested a cooler sensation of the linen fabrics with panama and twill weave, among all six fabrics investigated, the hemp–linen fabric with a plain weave seemed to be the optimal choice when a cool sensation was desired. Higher thermal conductivity values also suggested better heat transfer properties of the hemp–linen fabrics, and these were significantly influenced by the weave type.

This paper complements the limited studies investigating hemp and hemp–linen fabrics. Although the number of investigated weaves and hemp yarns was rather limited, the results are promising and clearly indicate the advantages of using hemp for improving physical and specific comfort properties of linen fabrics. Further research will extend the number of constructional parameters and type of hemp yarns and investigate their influence on additional fabric properties. Furthermore, 100% hemp fabrics will be developed, and their performance will be compared with linen or less sustainable counterpart fabrics.

## Figures and Tables

**Table 1:** Constructional parameters of the six fabrics differentiated by type of weft yarns (hemp or flax) and type of weave (plain, panama, and twill).

Fabric	Fabric ID	Weft Yarn	Warp Yarn	Weave	Weft Density (picks/cm)	Warp Density (ends/cm)
1	HEMP PLAIN	Hemp	Flax	Plain 1/1 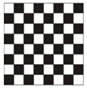	23	24
2	FLAX PLAIN	Flax	Flax		23	24
3	HEMP PAN	Hemp	Flax	Panama 2/2 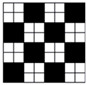	23	24
4	FLAX PAN	Flax	Flax		23	24
5	HEMP TWILL	Hemp	Flax	Twill 2/2 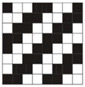	23	24
6	FLAX TWILL	Flax	Flax		23	24

**Table 2:** Fabric physical properties (mean ± STDEV).

Fabric ID	Mass per Unit Area (g·m^−2^)	Thickness (mm)	Bulk Density (kg·m^−3^)	Porosity (%)
HEMP PLAIN	193.2 ± 2.1	0.52 ± 0.01	375.2 ± 10.6	75.1 ± 0.7
HEMP PAN	181.3 ± 2.8	0.54 ± 0.01	338.4 ± 9.7	77.5 ± 0.6
HEMP TWILL	184 ± 3.2	0.55 ± 0.01	333.4 ± 8.6	77.8 ± 0.6
FLAX PLAIN	199.7 ± 1.7	0.51 ± 0.01	392 ± 10.8	74.5 ± 0.7
FLAX PAN	188.9 ± 1.9	0.55 ± 0.02	346.2 ± 9.7	77.5 ± 0.6
FLAX TWILL	188.9 ± 2.6	0.54 ± 0.01	350.1 ± 5.9	77.3 ± 0.4

**Table 3:** Results of a *t*-test (two samples with unequal variances) showing significant differences (*p*-value < 0.05) between the mechanical properties (i.e., load at break, elongation, and bending load) of the six fabrics depending on weft type (hemp/flax) and fabric direction (weft/warp).

Fabric ID		HEMP PLAIN	FLAX PLAIN	HEMP PAN	FLAX PAN	HEMP TWILL	FLAX TWILL
Influence of weft type (hemp versus flax) on fabrics with same weave (PLAIN, PAN, and TWILL)							
Load at break	Weft	0.97		0.049		0.00078	
	Warp	0.23		0.25		0.57	
Elongation	Weft	0.05		0.0036		0.00013	
	Warp	<0.05		<0.05		<0.05	
Bending load	Weft	<0.05		<0.05		<0.05	
	Warp	0.33		0.84		0.32	
Influence of direction (weft versus warp)							
Load at break		0.003	0.002	0.05	0.19	0.23	0.69
Elongation		<0.05	<0.05	<0.05	<0.05	<0.05	<0.05
Bending load		<0.05	<0.05	<0.05	<0.05	<0.05	<0.05

**Table 4:** Number of abrasion cycles required to cause breakdown of the test specimen.

	HEMP PLAIN	HEMP PAN	HEMP TWILL	FLAX PLAIN	FLAX PAN	FLAX TWILL
Number of rubbing cycles	5000	4000	6000	5000	4000	6000

**Table 5:** Results of a *t*-test (two samples with unequal variances) showing significant differences (*p*-value < 0.05) between comfort properties (i.e., air permeability, maximum heat flux Qmax, and thermal conductivity TCC) of the six fabrics depending on weft type (hemp/flax).

Fabric ID	HEMP PLAIN	FLAX PLAIN	HEMP PAN	FLAX PAN	HEMP TWILL	FLAX TWILL
Influence of weft type (hemp versus flax) for fabrics with same weave (PLAIN, PAN, and TWILL)						
*p*-value air permeability	<0.05		0.18		0.015	
*p*-value Qmax	0.004		0.007		0.03	
*p*-value TCC	0.33		0.12		0.21	

**Table 6:** Moisture management properties (mean ± STDEV) of the six fabrics.

	Unit	HEMP PLAIN	HEMP PAN	HEMP TWILL	FLAX PLAIN	FLAX PAN	FLAX TWILL
Wetting time top	s	3.7 ± 1.4	42.2 ± 60.3	26.5 ± 52.3	4.2 ± 2.2	4 ± 0.8	5.2 ± 0.7
Wetting time bottom	s	3.8 ± 1.2	4.8 ± 2.4	5.8 ± 5.3	5.5 ± 1.8	4.5 ± 0.7	5.7 ± 2
Top absorption rate	%/s	34.3 ± 12.2	14.6 ± 15.8	28.9 ± 34.2	29.2 ± 18.2	7.3 ± 0.9	18 ± 13.2
Bottom absorption rate	%/s	58.8 ± 17.5	55 ± 15.1	50 ± 20.1	29.5 ± 16.5	45.4 ± 21.8	51.5 ± 16.6
Top max wetted radius	mm	18.6 ± 3.8	9.2 ± 8	8 ± 5.7	5.6 ± 1.8	6.7 ± 2.6	6.7 ± 2.6
Bottom wetted radius	mm	18.6 ± 3.8	13.3 ± 4.1	12 ± 4.5	6.3 ± 2.3	7.5 ± 2.7	7.5 ± 2.7
Top spreading speed	mm/s	2.7 ± 0.8	1.7 ± 1.6	2.1 ± 1.4	1.5 ± 0.8	1.3 ± 0.3	1.1 ± 0.3
Bottom spreading speed	mm/s	2.6 ± 0.8	1.9 ± 1.1	2.3 ± 0.9	1.1 ± 0.6	1.2 ± 0.1	1.1 ± 0.3
Accumulative one-way transport index (R)	%	164.2 ± 84.6	385.4 ± 248.7	425.9 ± 172.7	429.6 ± 315.2	765 ± 177	598.5 ± 123
OMMC	-	0.5 ± 0.1	0.6 ± 0.1	0.7 ± 0.2	0.5 ± 0.2	0.6 ± 0.1	0.6 ± 0.1

OMMC—overall moisture management capability.

## Data Availability

Data are contained within the article.
